# Antioxidant and Photoprotective Effects of Blanch Water, a Byproduct of the Almond Processing Industry

**DOI:** 10.3390/molecules181012426

**Published:** 2013-10-09

**Authors:** Giuseppina Mandalari, Teresita Arcoraci, Maria Martorana, Carlo Bisignano, Luisa Rizza, Francesco Paolo Bonina, Domenico Trombetta, Antonio Tomaino

**Affiliations:** 1 Dipartimento di Scienze del Farmaco e dei Prodotti per la Salute, University of Messina, Viale Annunziata, Messina 98100, Italy; 2 Model Gut Platform, Institute of Food Research, Norwich Research Park, Colney Lane NR4 7UA, Norwich, UK; 3 Dipartimento di Scienze Biologiche ed Ambientali, University of Messina, Sal. Sperone, Messina 98100, Italy; 4 Dipartimento of Pharmaceutical Science, Faculty of Pharmacy, University of Catania, Viale Andrea Doria, Catania 95125, Italy

**Keywords:** blanch water, byproduct, photoprotective, antioxidant, polyphenols

## Abstract

The aim of the present work was to evaluate the antioxidant and photoprotective effect of blanch water (BW), a byproduct of the almond processing industry. The polyphenolic content of a BW extract, the level of proanthocyanidins and the vanillin index determination were determined. The antioxidant activity and the radical scavenging activity of the BW were evaluated by a range of *in vitro* tests. The *in vivo* photoprotective effect was investigated using a formulation containing 2% of the BW extract on skin erythema induced by acute UV-B exposure in twelve volunteers. Results confirmed the presence of added-value antioxidant compounds in the industrial BW extract, and the most representative compounds were naringenin-7-*O*-glucoside and kaempferol-7-*O*-rutinoside. The proanthocyanidin content was 71.84 ± 5.21 cyanidin equivalents/g of BW extract. The good antiradical activity of the BW extract was demonstrated in both the DPPH^•^ test and in the Reducing Power test. The percentage inhibition of erythema obtained using a formulation of BW was 50.48, value clearly demonstrating an effect against photooxidative damage *in vivo*.

## 1. Introduction

In recent human studies, almond consumption was shown to have beneficial effects on blood glucose levels in individuals with type-2 diabetes and prediabetes, with statistically-significant improvements in fasting levels of glucose, insulin, insulin sensitivity and LDL-cholesterol [1,2]. Evidence from observational reports have also indicated that phytochemicals may be related to the beneficial effects associated with almond consumption, reducing the risk of several chronic conditions and certain forms of cancer [3,4]. A number of studies have reported on the distribution of flavonoids and phenolics in almond skins [5,6]. Almond skin is recognised as a useful ingredient for the control of oxidative processes in food products and as a functional food ingredient in its own right. We have previously identified the flavonoids (flavanols, flavonols and flavanones) and phenolic acids present in almond skins and demonstrated that they are bioaccessible in the upper gastrointestinal (GI) tract and therefore potentially available for absorption during digestion [5,7]. The most represented flavonoids present in almond skins are (+)-catechin, (‒)-epicatechin, kaempferol, and isorhamnetin, the latter as 3-*O*-rutinoside or 3-*O*-glucoside [5]. Blanched almond skins, industrially removed from the nut by hot water blanching, constitute 4-8% of the total shelled almond weight and are considered byproducts, which if not processed further, are discarded as waste. As expected, blanching results in a substantial loss of polyphenols and antioxidant activity to the blanch water [5,6]. The almond processing industries are interested in the valorisation of these byproducts which at present are mainly used in cattle feed [8] and in gasification plants to produce energy [9].

The protective effect of plant polyphenols from damage produced by reactive oxygen species (ROS) including exposure to ultraviolet (UV) radiation has been previously reported [10,11]. A range of natural compounds has been investigated for their photoprotective effect, exerted both when ingested through the diet and if topically applied [12,13].

The aims of the present work were to investigate the antioxidant properties of an industrial extract of almond blanch water (BW) and evaluate its potential use in topical photoprotective formulation to improve skin health. On the basis of the *in vitro* antioxidant data, a topical formulation containing BW extract was tested *in vivo* for its ability to reduce UV-B-induced skin erythema. This test is considered the most representative model to investigate skin damage *in vivo* post-acute UV-exposure [14,15].

## 2. Results and Discussion

### 2.1. Polyphenolic Content in Blanch Water

Table 1 shows the polyphenolic content in BW, expressed as μg/g extract of phenolic acids, flavan-3-ols, flavanones and flavonols. As previously reported [5], the most representative compounds identified were naringenin-7-*O*-glucoside and kaempferol-7-*O*-rutinoside, followed by catechin and 5-hydroxybenzoic acid. Neither quercetin nor kaempferol were found in BW, mainly due to their poor water solubility.

**Table 1:** Quantitative analysis of phenolics in the BW extract. Results are expressed as mean±SD of three independent samples.

Phenol	BW (µg/g extract)
**Phenolic acids**	778.71 ± 65.35
Protocatechuic acid	73.19 ± 6.35
*p*-Hydroxybenzoic acid	542.37 ± 45.81
Chlorogenic acid	84.32 ± 7.64
Vanillic acid	63.54 ± 4.51
*trans p*-Coumaric acid	15.29 ± 1.04
**Flavan-3-ols**	1069.50 ± 79.67
*Aglycones*	*1069.50 ± 79.67*
Catechin	693.41 ± 51.49
Epicatechin	376.09 ± 28.18
**Flavanones**	842.65 ± 82.94
*Aglycones*	*69.99 ± 5.64*
Eriodictyol	16.27 ± 1.24
Naringenin	53.72 ± 4.40
*Glycosides*	*820.23 ± 77.30*
Eriodictyol-7-*O*-glucoside	4.85 ± 2.71
Naringenin-7-*O*-glucoside	815.38 ± 74.59
**Flavonols**	1089.54 ± 89.81
*Glycosides*	*1089.54 ± 89.81*
Quercetin-3-*O*-rutinoside	79.43 ± 6.21
Quercetin-3-*O*-glucoside	7.13 ± 0.62
Kaempferol-3-*O*-rutinoside	803.54 ± 65.03
Kaempferol-3-*O*-glucoside	33.73 ± 2.76
Isoramnetin-3-*O*-rutinoside	42.12 ± 3.57
Isoramnetin-3-*O*-glucoside	123.59 ± 11.62

Although a similar qualitative profile has been reported by other authors [6], it is not possible to make meaningful comparisons due to differences in variety, year of production, environmental conditions and extraction methods [16]. Furthermore, Milbury *et al*. [6] designed experimental blanching conditions mimicking industrial processing, whereas the BW used in the current study was industrially produced. These data confirmed the presence of added-value antioxidant compounds in the industrial BW extract, which may be used as dietary antioxidant ingredients.

Proanthocyanidins have been reported to possess a broad spectrum of biological, pharmacological and therapeutic properties related to their radical scavenging and antioxidant activities [17,18,19]. In particular, the presence of one or more catechol moieties in their structure seems to be a key factor in determining the scavenging activity. In our study, the content of proanthocyanidins in BW extract was 71.84 ± 5.21 CyE/g of extract (Table 2). The vanillin index provides an estimate of the number of C-6 and C-8 free of both catechins and proanthocyanidins. Proanthocyanidins are slightly less reactive than the catechins only when at least one of the two sites, C-6 and C-8, is free. This index decreases with the increase in polymerization degree, because many of the C-6 and C-8 positions are involved in the polymerization step. We measured a vanillin index of 29.45 ± 2.69 mg of catechin equivalents/g of BW extract. The polymerization index is the result of the vanillin assay divided by the proanthocyanidin assay; this ratio provides a rough estimate of the degree of polymerization of the flavonols. The low value (0.41) of polymerization index in the BW extract calculated in the present work indicates that the BW extract has large amount of polymeric tannins. Our results are different compared to the values reported by Pérez-Jiménez and Lluís Torres [20], possibly because the BW extract used in the current work, which was not submitted to any extraction process with organic solvent, did not contain extractable proanthocyanidins.

**Table 2:** Content of proanthocyanidins, flavanols reactive to vanillin (vanillin index) and polymerization index determined in the blanch water (BW) extract. Results are expressed as mean ± SD of three independent determinations.

Component	BW
Proanthocyanidins *mg CyE/g extract ^a^*	71.84 ± 5.21
Vanillin index *mg CatE/g extract ^b^*	29.45 ± 2.69
Polymerization Index	0.41

^a^ CyE: Cyanidin chloride Equivalents; ^b^ CatE: Catechin Equivalents.

### 2.2. Antioxidant Activity of Blanch Water

The antioxidant and radical scavenging properties of the polyphenolic compounds present in BW has been demonstrated using a range of tests (Table 3).

**Table 3:** Antioxidant activity of the blanch water (BW) extract measured by means of different *in vitro* tests. Data are expressed as mean±SD of three independent experiments.

Assay	Unit	BW
Folin-Ciocalteau test	mg GAE/g	90.28 ± 5.47
DPPH test	^a^ SE_50_ (µg)	132.82 ± 12.02
Reducing Power test	^b^ mmoles AAE/g	2.02 ± 0.11
β-Carotene blanching test	^c^ IC_50_ (µg/mL)	232.86 ± 21.45
UV-IP test	^d^ IC_50_ (µg/mL)	531.11 ± 44.91

^a^ mg of blanch water extract needed to scavenge 50 mmoles of DPPH. ^b^ mmoles of ascorbic acid equivalent/g of extract. ^c^ mg/mL of extract needed to inhibit by 50% β-carotene bleaching. ^d^ mg/mL of extract needed to inhibit by 50% MDA production.

The total phenolic content expressed as mg of gallic acid equivalents (GAE) per g of BW extract was 90.28 ± 5.47. This value is directly comparable with our previous investigation [5] and significantly lower than the values reported by Milbury *et al*. [6], who found between 50.3 mg GAE and 153.9 mg GAE from blanching 100 g of fresh natural almonds. The good antiradical activity of the BW extract showed in the DPPH∙ test (expressed as SE_50_, amount needed to scavenge 50 µmoles of the initial DPPH∙ solution) and in the Reducing Power test (expressed as mmoles ascorbic acid equivalents per g of BW extract) confirmed the higher antioxidant capacity *in vitro* of almond polyphenols compared to vitamins and carotenoids [21].

Both the Folin-Ciocalteau method and the Reducing Power test are electron/transfer based assays, performed in alkaline and acid conditions, respectively. The acidic pH may reduce the activity of phenolic antioxidants *in vitro* due to protonation, whereas the alkaline pH could enhance the antioxidant potential due to dissociation [22]. Therefore, a range of tests is always warranted to evaluate the antioxidant potential of polyphenolic compounds *in vitro*. The activity found in the BW extract by the Folin-Ciocalteau method and the Reducing Power test was higher compared to pistachio seeds and lower than the values obtained with pistachio skin [11]. The results obtained with the β-carotene bleaching test and the UV-IP test confirmed that the antioxidant compounds present in BW were able to protect from lipid peroxidation induced by heating or UV-C light.

### 2.3. *In Vivo* Photoprotective Effect of Blanch Water

**Figure 1:**
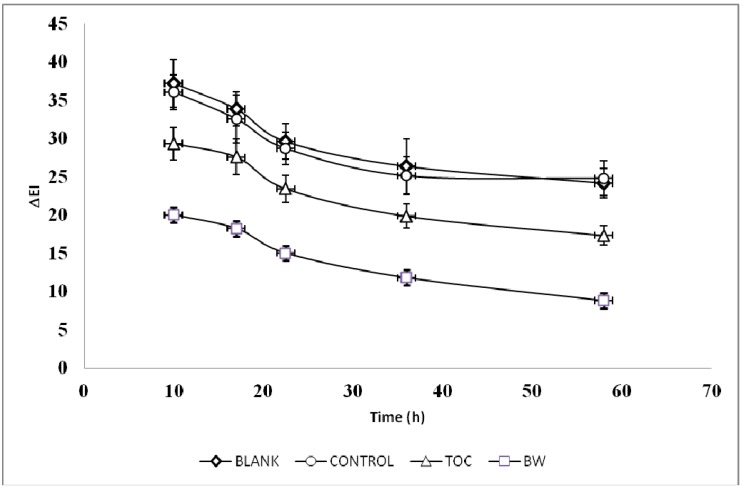
Typical trend of erythema index variations (*Δ*EI) *versus* time for one subject. Formulations containing 2% of the active ingredients under study (blanch water extract, BW, or tocopheryl acetate, TOC) or containing no active ingredient (blank) were applied to the skin after UV-B exposure. No treated skin sites were used as control.

On the basis of the *in vitro* antioxidant data, the BW extract was used as topical photoprotective agent in human volunteers suffering from skin erythema induced by acute UV-B exposure. Figure 1 shows the typical trend of erythema index variations (*Δ*EI) *versus* time for one subject when applying a formulation containing either the BW extract (2%) or tocopheryl acetate (TOC, 2%).

Both active ingredients were able to better protect the skin against UV-B erythema compared to the blank formulation and the control skin site. The AUC_0-58_ values, which refer to the change of the UV-B erythema over time for each volunteer, were slightly lower with TOC formulation compared to the BW extract (*p* < 0.05) (Table 4). These data are well comparable with the results obtained using an extract of pistachio seed and pistachio skin, demonstrating the potential beneficial effect of nut polyphenols on skin health [11]. Other plant extracts have successfully been tested as photoprotective agents [13,15,23,24,25,26,27,28,29] and their skin healthy effects have been attributed to the high content of phenolic compounds.

**Table 4:** AUC_0–58_ values obtained by applying either the blanch water extract (BW), tocopheryl acetate (TOC) or blank formulations to UV-B-exposed skin sites. Data are expressed as mean ± SD and analyzed by Student’s t test for unpaired data.

Subject	Control	Blank	TOC	BW
A	1383.27 ± 120.59	1365.41 ± 115.67	1126.38 ± 66.12	654.68 ± 50.67
B	1522.18 ± 102.40	1356.08 ± 100.09	954.23 ± 73.64	700.36 ± 55.24
C	1125.35 ± 97.97	1296.34 ± 95.67	1115.45 ± 69.58	765.68 ± 49.29
D	1248.05 ± 120.26	1456.44 ± 134.25	1048.11 ± 75.15	643.28 ± 50.71
E	1392.61 ± 124.65	1108.87 ± 87.43	1084.65 ± 79.26	696.03 ± 45.38
F	1275.87 ± 100.65	1284.60 ± 102.58	1035.44 ± 65.37	624.95 ± 47.08
G	1396.14 ± 115.38	1326.94 ± 118.05	973.12 ± 70.25	644.76 ± 56.16
H	1471.85 ± 121.37	1285.67 ± 112.82	1219.12 ± 76.41	545.84 ± 39.88
I	1329.53 ± 104.41	1574.24 ± 136.57	1065.21 ± 81.36	712.59 ± 42.79
L	1507.39 ± 99.37	1429.41 ± 126.48	941.72 ± 62.74	907.33 ± 57.29
M	1436.27 ± 124.84	1230.50 ± 95.66	1119.35 ± 80.54	650.82 ± 45.44
N	1284.08 ± 104.73	1236.77 ± 122.5	1023.68 ± 68.25	560.61 ± 48.36
mean	1364.38	1329.27	1058.87	675.57
± S.D.	116.93	120.64	80.75	95.29
PIE (%)	- - -	- - -	22.39 ^a^	50.48 ^a,b^

^a^ *p* < 0.05 *vs.* Control, ^b^ *p* < 0.05 *vs.* TOC.

**Figure 2:**
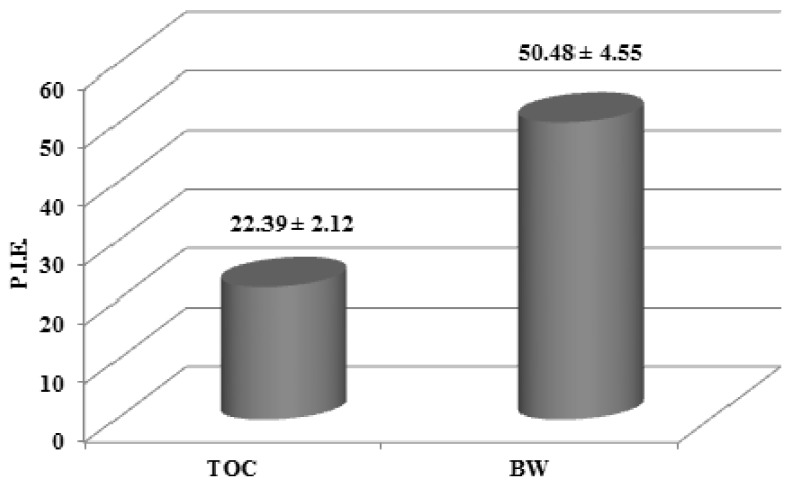
Percentage inhibition of erythema (PIE) values obtained after skin application of formulations containing either the blanch water extract (BW, 2%) or tocopheryl acetate (TOC, 2%), calculated *vs*. blank samples.

The percentage inhibition of erythema (PIE) values obtained using formulations of either BW or TOC were 50.48 and 22.39, respectively (Figure 2). These results clearly demonstrated an effect of BW against photooxidative damage *in vivo*.

## 3. Experimental

### 3.1. Preparation of the Blanch Water (BW) Extract

BW, after thawing, was brought to room temperature and maintained under continuous stirring. An aliquot (20 mL) was centrifuged at 4,200 × *g* for 5 min (Minifuge 2; Heraeus-Christ, Osterode, Germany), the supernatant was dried by a rotary evaporator (R-205; Büchi, Flawil, Switzerland) and the obtained residue (73.10 mg) was stored at −20 °C until further use. For all experiments, the BW extract was dissolved in the minimum volume of a mixture methanol (purity ≥ 99.9%; Carlo Erba, Milan, Italy)/water (2/1).

### 3.2. HPLC Analysis of Phenolics and Flavonoids

The qualitative/quantitative determination of phenolics and flavonoids in BW was carried out using a Shimadzu high performance liquid chromatography system equipped with a UV–vis photodiode-array detector (DAD, SPD-M10Avp) and a fluorescence detector (1046A; Hewlett Packard). The apparatus was controlled by a control system (SCL-10Avp) equipped with an LC pump (LC-10 ADvp) and an auto-injector (SIL-10ADvp). The chromatographic separation was obtained by a 5 mm ODS3 reversed-phase Prodigy column (250 mm × 4.6 mm; Phenomenex, Inc., Torrance, CA, USA) with solvent A (water/acetic acid (HPLC grade, purity ≥99.5%; Rudi Pont, Carlo Erba, Italy), 98/2, v/v), solvent B (water/acetonitrile (HPLC PLUS grade, purity ≥99.9%; Carlo Erba)/acetic acid, 73/25/2, v/v/v) and solvent C (acetonitrile) as mobile phase [3]. The gradient program started with 100% A to reach 20% A and 80% B at 55 min, 10% A and 90% B at 57 min, 100% B at 90 min and 100% C at 105 min. The flow rate was 1 mL/min, and the column was thermostatically controlled at 25 °C. The extract was filtered through a 0.2 µm filter (nylon; Millipore Corporation, Milford, MA, USA) and the injection volume was 75 µL. All analyses were carried out in triplicate. Identification of compounds was carried out by comparing their spectra and retention times with those of external standards. Detection was performed at 270 nm for hydroxybenzoic acids and flavanones and at 370 nm for flavonols. The UV spectra of the different compounds were recorded from 240 to 400 nm. The wavelengths used for fluorescence detection of flavan-3-ols were: λ_ex_: 276 nm, λ_em_: 316 nm. Data acquisition was performed using Class-VP5 Chemstation software (Shimadzu, Kyoto, Japan). All flavonoid and other phytochemical standards (flavanones glycosides and aglycones, flavone glycosides, phenolic acids) were obtained either from Sigma–Aldrich (Poole, UK) or Extrasynthese (Genay, France).

### 3.3. Levels of Proanthocyanidins

The method used for determining the content of proanthocyanidins is based on their transformation into anthocyanidins, leading to a shift of colour towards the red zone in a warm and acid environment [26]. An aliquot (50 mg) of the extract, diluted (approximately 10-20 times) with 0.05 M H_2_SO_4_, was loaded onto a conditioned Sep-Pak C_18_ (Waters, Milford, MA, USA), which was washed with 2 mL of 5 mM H_2_SO_4_ and purged with air. The fraction rich in proanthocyanidins was eluted with 3 mL of MeOH and collected into a 50 mL flask shielded from light (aluminium foil) and containing 9.5 mL absolute EtOH. The mixture was added with 12.5 mL of FeSO_4_∙7H_2_O in concentrated HCl (300 mg/L) and the flask was then placed in a boiling water bath and refluxed for 50 min, after which it was rapidly cooled by immersion in cold water (20 °C). After 10 min, the absorbance at 550 nm was registered on a UV-Visible spectrophotometer (UV-1601 UV-visible; Shimadzu). To account for the basal content of anthocyanins present in the sample, the corresponding absorbance value of each extract prepared under the same conditions, but cooled in ice and not warmed, was subtracted to obtain the net absorbance value. Under such conditions, the average yield was 20% and the proanthocyanidin concentration can be conventionally expressed as 5 times the amount of cyanidin formed, by means of a calibration curve with cyanidin chloride (ε = 34,700 according to Di Stefano, Cravero, and Gentilini [30]). Each determination was performed in triplicate.

### 3.4. Vanillin Index Determination

The concentration of polyphenols reactive to vanillin in a highly acid environment was determined according to the method described by Margheri and Falcieri and by Tomaino *et al*. [31,32]. Vanillin is a relatively stable aldehyde at high H_2_SO_4_ concentrations; it reacts with free carbons C6 and C8 of flavan-3-ols, leading to the formation of a red complex with maximum absorbance at 500 nm. Briefly, 2 mL of the extract, diluted with 0.5 M H_2_SO_4_ to obtain a final reading between 0.2 and 0.4 AU, were loaded onto a conditioned Sep-Pak C_18_ (Waters Corporation). The column was washed with 2 mL of 5 mM H_2_SO_4_, purged with air and eluted with 5 mL of MeOH into a test tube. One milliliter of the methanolic eluate was placed in a test tube (shielded from light), together with 6 mL of 4% vanillin in MeOH and immersed in a water bath at 20 °C for 10 min. After cooling, 3 mL of concentrated HCl were added. After 15 min, the absorbance of the pigment was read on a Shimadzu UV-1601 UV-visible spectrophotometer at 500 nm against a blank prepared under the same conditions and containing only MeOH. Results were expressed as mg of CatE for g of extract. Each determination was performed in triplicate.

### 3.5. Evaluation of Antioxidant Activity

#### 3.5.1. Folin-Ciocalteau Method

The antioxidant capacity of the BW extract was determined by the Folin-Ciocalteau reagent [29]. Total phenol content was expressed as mg of gallic acid equivalents (GAE)/g of extract. Each determination was performed in triplicate and repeated at least three times.

#### 3.5.2. Radical Scavenging Activity

The anti-radical activity of BW was determined using the stable 1,1-diphenyl-2-picrylhydrazyl radical (DPPH^•^) (Sigma, Milan, Italy) and the procedure previously described [28]. In its free radical form, DPPH^•^ has an absorption band at 517 nm which disappears upon reduction by an anti-radical compound. Absorbance at 517 nm was measured on a Shimadzu UV-1601 UV–vis spectrophotometer 20 min after starting the reaction. The DPPH^•^ concentration in the reaction medium was calculated from a calibration curve analyzed by linear regression. Each determination was carried out in triplicate and repeated at least three times. Results were expressed as mg of extract needed to scavenge 50 mmoles of initial DPPH concentration (SE 50).

3.5.3. β-Carotene Bleaching Test

This assay was carried out according to the method of Aidi Wannes *et al*. [33] with some modifications. To prepare a stock solution of a *β*-carotene-linoleic acid mixture, 1 mg of β-carotene (purity ≥ 97%; Sigma) was dissolved in 10 mL of chloroform (HPLC grade, purity ≥ 97%; Carlo Erba), and then 5 mL of this solution were added to 40 μL of linoleic acid (purity ≥ 99%; Sigma) and 400 μL of Tween 40 (Sigma). Chloroform was removed using a rotary evaporator at 40 °C for 5 min and then 100 mL of distilled water were slowly added to the residue to form an emulsion. Five milliliters of the emulsion were added to 200 μL of methanol/water (2/1) solution containing the extract to be studied at different concentrations (0.25–10 mg/mL); the same volume of the solvent alone (methanol/water, 2/1) was used in control samples. The absorbance was immediately measured (t = 0 min) at 470 nm against a blank, consisting of an emulsion without β-carotene. The sample was then placed in a water bath at 50 °C and the oxidation of the emulsion was monitored by measuring absorbance at 470 nm 120 min after the beginning of the reaction. The percentage of inhibition respect to the control was calculated as follows:

(1) % of inhibition = [(A_t_ − C_t_)/C_0_ − C_t_]] × 100

where A_t_ and C_t_ are the absorbances measured for the tested sample and the control sample, respectively, at t = 120 min, and C_0_ is the absorbance value for the control sample measured at t = 0 min. Each determination was carried out in triplicate and repeated at least three times. Results were expressed as mg/mL of extract needed to inhibit β-carotene bleaching by 50% (IC_50_).

#### 3.5.4. Reducing Power Test

The reducing power of the blanch water extract was determined according to the method of Oyaizu [34] as amended by Martorana *et al*. [11]. In this assay, the yellow colour of the test solution turns into green/blue whose intensity depends on the reducing power of the antioxidants present in the solution, which cause the reduction of the Fe^3+^/ferricyanide complex to the ferrous form; therefore, Fe^2+^ can be monitored by the measurement of the absorbance at 700 nm.

Briefly, 200 μL of methanol/water (2/1) solutions containing BW extract at a concentration able to give a final reading between 0.3 and 0.7 AU were mixed with 0.5 mL of 0.2 M sodium phosphate buffer (pH 6.6) (sodium phosphate dibasic heptahydrate (Na_2_HPO_4_, ACS Reagent grade, purity 98%–102%; Sigma), sodium phosphate monobasic monohydrate (NaH_2_PO_4_·H_2_O, ACS Reagent grade, purity 98%–102%; Sigma) and 0.5 mL of 1% K_3_Fe(CN)_6_ (purity 99%; Sigma), and then incubated in a water bath at 50 °C for 20 min. 0.5 mL of 10% trichloroacetic acid (TCA, Sigma) was then added to the mixture which was centrifuged at 8,300 × *g* for 10 min. The supernatant (0.5 mL) was mixed with 0.5 mL of distilled water and 0.1 mL of 0.1% ferric chloride solution (purity ≥ 99.99%; Sigma). The intensity of the blue-green colour was measured at 700 nm. Each determination was performed in triplicate and repeated at least three times.

Under the same conditions described above, different concentrations of ascorbic acid (AA, Sigma) were tested in order to measure their reducing power. A calibration curve of Prussian Blue (Sigma) (concentration range 10–150 μM), dissolved in a mixture of water, phosphate buffer, TCA and methanol (71.48/13.37/13.37/1.78), was used to calculate the number of nmoles of Prussian Blue formed by the reaction between AA and Fe^3+^. The number of Fe^2+^ nmoles was calculated as follows:

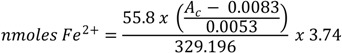
(2)
where 55.8 is the atomic weight of iron; A_C_ is the absorbance at 700 nm of the reaction mixture containing AA; 0.0083 and 0.0053 are intercept and slope values of Prussian Blue equation, respectively; 329.196 is the molecular weight of Prussian Blue; 3.74 is the dilution factor.

Thus, plotting the number of AA nmoles employed *vs* the number of formed Fe^2+^ nmoles, a straight line was obtained, which was used to calculate the reducing power of the tested extract. The results were expressed as mmoles of AA equivalents (AAE) per gram of extract.

#### 3.5.5. UV-induced Peroxidation in Liposomal Membranes (UV-IP Test)

The protective effect of the BW extract against UVC-induced peroxidation was evaluated on phosphatidylcholine (PC) multilamellar vesicles [35]. Briefly, a volume (950 μL) of liposome suspension containing 10 mg/mL of PC (from egg yolk type XI-e; Sigma) (in a glass flask with a 3 cm^3^ exposure surface area) was exposed to UV radiation from a 15 W Philips germicidal lamp (254 nm) for 1.5 h. Exposure was given at 10 cm from the lamp, and the experiment was carried out at 37 °C. Fifty microliters of a methanol/water (2/1) mixture containing the extract to be tested at different concentrations were added to the system; an equal volume (50 μL) of the vehicle alone was added to control tubes. Malondialdehyde (MDA) concentration formed in the mixtures following UV-C irradiation was measured using a colorimetric assay. In this assay, N-methyl-2-phenylindole (purity 99%; Sigma) reacts with MDA to give a stable chromophore with a λ_max_ at 586 nm. To calculate the amount of MDA formed in the samples, a calibration curve was prepared by submitting methanolic solutions of 1,1,3,3-tetramethoxypropan (Sigma), at different concentrations, to the same procedures described above. All determinations were carried out in triplicate and repeated at least three times. The results were expressed as concentration of the extract needed to inhibit MDA formation by 50% (IC50) with respect to the control.

### 3.6. *In Vivo* Study on Photoprotective Activity

#### 3.6.1. Preparation of Topic Formulations

To evaluate the photoprotective effect of the extract under investigation *in vivo*, we used a formulation containing either 2% BW extract or 2% tocopheryl acetate (TOC) (as a reference ingredient). The composition of each formulation is reported in Table 5. Each oil/water (O/W) emulsion was prepared by slowly adding the aqueous phase to the oily phase and by blending the surfactants under continuous agitation, at 70 °C. The mixture was stirred until cool, forming the emulsion formulation. Moreover, a formulation without active ingredients was used in the study (blank). PPG-15 stearyl ether, steareth 2, steareth 21, isohexadecane\PPG-15 stearyl ether; cetylstearylic acid; xantan gum; undebenzophenon were bought from Croda (Milan, Italy); stearic acid was purchased from Fluka.

**Table 5:** Composition of the topical formulations containing the Blanch Water (BW) extract or tocopheryl acetate as reference substance (TOC), or without active ingredients (Blank).

Formulation	Oil phase	Acqueous phase	*Surfactants and structurizing agents*
Blank	PPG-15 stearyl ether (8 g); isohexadecane\PPG-15 stearyl ether (4 g)	Distilled water (76.7 g)	*Steareth 2 (3.5 g)*
			*Steareth 21 (2.5 g)*
			*Stearic acid (2.5 g)*
			*Cetylstearylic acid (2.1 g)*
			*Xantan gum (0.3 g)*
			*Undebenzophenone (0.4 g)*
TOC	PPG-15 stearyl ether (7 g); isohexadecane\PPG-15 stearyl ether (3 g); tocopheryl acetate (2 g)	Distilled water (76.7 g)	*Steareth 2 (3.5 g)*
			*Steareth 21 (2.5 g)*
			*Stearic acid (2.5 g)*
			*Cetylstearylic acid (2.1 g)*
			*Xantan gum (0.3 g)*
			*Undebenzophenone (0.4 g)*
BW	PPG-15 stearyl ether (8 g); isohexadecane\PPG-15 stearyl ether (4 g)	Distilled water (74.7 g) Blanch water extract (2 g)	*Steareth 2 (3.5 g)*
			*Steareth 21 (2.5 g)*
			*Stearic acid (2.5 g)*
			*Cetylstearylic acid (2.1 g)*
			*Xantan gum (0.3 g)*
			*Undebenzophenone (0.4 g)*

#### 3.6.2. Instruments

Skin erythema was induced by UV-B irradiation using a UVM-57 ultraviolet lamp (UVP, San Gabriel, CA, USA). This source emits radiation in the range of 290–320 nm with an output peak at 302 nm. The flux rate measured at the skin surface was 0.80 mW cm^−2^. For each subject the minimal erythema dose (MED) was determined and an irradiation dose corresponding to double the MED was used throughout the study. UV-B-induced skin erythema was monitored by using a reflectance visible spectrophotomer X-Rite model 968 (X Rite Inc., Grandville, MI, USA), having 0° illumination and 45° viewing angle, calibrated and controlled as previously reported [27]. Reflectance spectra were obtained over the wavelength range 400–700 nm using illuminant C and 2° standard observer.

#### 3.6.3. Subjects

*In vivo* experiments were performed on twelve healthy volunteers of skin types II and III, aged 25–35 years. The subjects were recruited after medical screening which included a health questionnaire and physical examination of the application sites. Subjects exhibiting features which might interfere with the evaluation, such as sunburn, skin lesions or abnormal sensitivity to sunlight, or taking medication at the time of the study, were excluded from the study. The volunteers were fully informed about the nature of the study, and about the products and procedures involved, and gave their written consent. Each subject was rested for 15 min before the experiments and room conditions were set at 22 ± 2 °C and 40%–50% relative humidity. Two research assistants were responsible for subject recruitment and data collection.

#### 3.6.4. Protocol

For each subject recruited in the experiment, eight sites in the ventral surface of the forearm were defined using a circular template (1 cm^2^) and demarcated with permanent ink. Baseline skin assessment was performed by reflectance spectrophotometry in all sites. Each site was exposed to UV-B irradiation, after which 200 mg of each formulation (containing either BW or TOC, or the blank) were spread uniformly on the sites by means of a solid glass rod. For each forearm, two of the ten sites were used as control (no drug treatment). Afterwards, each skin site was occluded for 3 h, using Hill Top chambers (Hill Top Research, Inc., Cincinnati, OH, USA), to prevent any loss of the material from the skin surface. After the occlusion period the chambers were removed and the skin surfaces were washed to remove the formulation and allowed to dry for 15 min, after which the induced erythema was monitored for 58 h by reflectance spectrophotometry. Since erythema is due to an increment of blood supply in the subpapillary plexus of the skin, erythema index (E.I.) values were calculated by subtracting the logarithm of inverse reflectance (log 1/R) values of 510 nm and 610 nm (mainly due to melanin absorption), from the sum of log 1/R values of 540, 560 and 580 nm, which represents the wavelengths of hemoglobin absorption peaks (Equation 3) [27]:

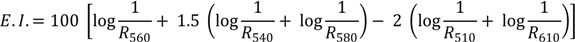
(3)


To evaluate the time course of skin erythema, E.I. baseline values (before UV-B irradiation) were subtracted from the E.I. values obtained after UV-B exposure at each time point, to calculate ΔE.I. values. For each site, ΔE.I. was plotted versus time and the area under the curve (AUC) was computed using the trapezoidal rule to obtain AUC0–58, a dimensionless index directly related to the degree of skin erythema. To better compare the efficacy of the tested formulations, the percentage inhibition of UV-B skin erythema (PIE) was calculated from formulation AUC0–58 values using the equation (Equation 4):

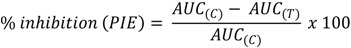
(4)
where AUC_(C)_ is the area under the response–time curve of the sites no treated (control) and AUC_(T)_ is the area under the response–time curve of the sites treated with the tested formulation. Statistical analysis of AUC values, expressed as means and standard deviations, was performed using unpaired Student’s t-test; p < 0.05 was considered to be significant.

## 4. Conclusions

Through our results we have demonstrated that blanch water, a byproduct of the almond processing industry, still contains high-value compounds which can be further utilized in the nutraceutical and pharmaceutical industries. A range of antioxidant tests has clearly demonstrated the radical scavenging capacity of blanch water and its potential use in conditions where an overproduction of free radicals occurs. The photoprotective effect of blanch water demonstrated *in vivo* has clearly highlighted an effect against photooxidative damage.
